# Self-efficacy of direct care workers providing care to older people in residential aged care settings: a scoping review protocol

**DOI:** 10.1186/s13643-021-01655-z

**Published:** 2021-04-10

**Authors:** Sumina Shrestha, Rayan Jafnan M. Alharbi, Christine While, Julie Ellis, Muhammad Aziz Rahman, Yvonne Wells

**Affiliations:** 1 Community Development and Environment Conservation Forum, Sindhupalchok, Nepal; 2 Australian Institute for Primary Care & Ageing, La Trobe University, Melbourne, Australia; 3 Department of Emergency Medical Service, Jazan University, Jazan, Saudi Arabia; 4 School of Nursing and Midwifery, La Trobe University, Melbourne, Australia; 5 School of Nursing and Healthcare Professions, Federation University, Melbourne, Australia

**Keywords:** Caring, Direct care workers, Residential aged care, Self-efficacy

## Abstract

**Background:**

Self-efficacy is developed through a person’s interaction with his/her physical and social environment. Self-efficacy in caring is an essential attribute of care workers to develop a positive attitude towards their clients, improve work performance, and enhance job satisfaction. Care workers’ self-efficacy may vary according to the context in which the care is being provided. Aged care is a multidimensional and challenging setting, and characteristics of aged care services are different from those of acute care services. The objective of this review is to give an overview of the self-efficacy of residential aged care workers in caring for older people and factors influencing their self-efficacy.

**Methods:**

The protocol for this review is based on the Joanna Briggs Institute Reviewer’s Manual for Scoping Review. A systematic search of the literature on electronic databases MEDLINE, PsycINFO, CINAHL, AgeLine, SCOPUS, and ProQuest Dissertations and Theses Global will be carried out using predefined search terms to identify relevant studies. This review will include studies that examined the self-efficacy of direct care workers in caring for older people living in residential aged care facilities. All primary studies irrespective of the study design will be included. Studies conducted to develop measures or studies with informal care workers or students as study participants will not be considered. Two reviewers will independently conduct title and abstract screening, full-text screening, and data charting. A third reviewer will resolve discrepancies, while the final decision for conflicting studies will be made by consensus within the review team. Descriptive statistics will be utilized to analyze the quantitative findings, and the result will be presented in narrative form accompanied by tables and charts. Content analysis will be carried to analyze the qualitative findings and will be presented in narrative form supported by illustrative quotations.

**Discussion:**

This study will be an important source of knowledge to policymakers and aged care providers to understand the self-efficacy of aged care workers to support and enhance their self-efficacy and thereby improve their caring behaviors towards their clients.

**Scoping review registration:**

Joanna Briggs Institute Systematic Review Register with the title “A scoping review of factors influencing caring efficacy of direct care workers providing care to older people”.

**Supplementary Information:**

The online version contains supplementary material available at 10.1186/s13643-021-01655-z.

## Background

Self-efficacy in caring refers to care workers’ beliefs or confidence in their ability to express caring behaviors and attitudes and to build compassionate relationships with their clients [1]. Confidence is an essential element to enhance competence to care and job satisfaction of care workers, as well as their perception towards managerial and practical aspects of care [1–3]. The stronger the individual’s confidence, the more active the efforts of an individual to perform a specific behavior or skill [4, 5]. Manojlovich [6] found a greater influence of self-efficacy of care workers in their job performance than the support services of an organization, such as information, resources, and opportunities. While increased self-efficacy in caring may improve care workers’ performance, low self-efficacy may adversely affect how these care workers carry out their duties [3, 6]. It is, therefore, essential to understand the self-efficacy of care workers and discuss factors that may influence their efficacy to demonstrate caring behaviors.

According to the Social Cognitive Theory, an individual’s interaction with the physical and social environment determines the self-efficacy of that person [7]. Human functioning is a product of the interaction of intrapersonal influences, the behavior one engages in, and the environment they grow and live in [5, 7]. Bandura [7] stressed that the self-efficacy of a person differs depending upon the individual’s physiological state, experiences, social relationships, and other circumstances he/she is living or working in. Therefore, the self-efficacy of care workers can vary according to the nature of their work and the clients they are caring for.

Characteristics of acute care hospitals and aged care facilities are very different [8]. In hospital settings, the aim of care workers or nursing staff is to care for patients suffering from acute health conditions or chronic health conditions with acute manifestations. Patients are expected to return to their place of residence following treatment. However, aged care services are provided to older people who are unable to live independently without assistance because of frailty and other age-related conditions [9]. The high level of dependency on the care provider, accompanied by increasingly deteriorating health conditions with complex and multiple diseases or disorders, makes older people a unique group of care recipients. Nursing care needs presented by older people in aged care is, therefore, often challenging and multidimensional, including physical, psychological, and social dimensions [8]. Hence, it is imperative to comprehend the self-efficacy of aged care workers and factors that may affect their confidence to care.

A qualitative study by Coates and Fossey found increased dilemma among care workers in the process of caregiving due to the lack of confidence in their role [10]. A study in Canadian long-term care home found a high level of perceived self-efficacy to provide palliative care among the care workers though there was a knowledge gap on palliative care [11]. Factors such as the age of the care worker, formal education, experience, and psychological empowerment were found to influence the confidence in delivering palliative care [12]. Studies by Caspar et al. have shown that perceived ability to provide care among care workers is influenced by their self-determination, quality of relationship with their immediate supervisors, educational opportunities, and recognition of the performance [13, 14]. Similarly, an intervention study found an increase in staff confidence in seeking support from their supervisors and dealing with end of life care symptoms after receiving training on compassion, communication, end of life symptoms, and care [15]. However, it did not find any improvement in staff’s belief in their ability to discuss death and dying with residents and their relatives post-training. Dementia training and peer support programs were found to enhance the self-efficacy of the care workers to care for the people living with dementia [16–18].

To understand the caring self-efficacy of aged care workers in general, we performed a literature search using broad search terms. However, we did not find any review article, which indicated a dearth of evidence synthesis in this area. Similarly, our preliminary search of the literature found that studies have utilized various instruments to measure self-efficacy, and interventions to address the self-efficacy of care workers are also not similar. Hence, a scoping review is planned to give an understanding of the self-efficacy of care workers in caring for older residents in residential aged care settings and identify factors that may influence care workers’ self-efficacy in caring.

### Review objectives

The objectives of this scoping review are as follows:

- Provide an overview of the self-efficacy of direct care workers in caring for older residents living in residential care settings.
- Identify factors influencing the self-efficacy of direct care workers in providing care to older residents in residential care settings.

## Methods

This protocol complies with the Preferred Reporting Items for Systematic reviews and Meta-Analyses for Protocols 2015 (PRISMA-P 2015) [19] (see Additional file 1) and is guided by the Joanna Briggs Institute (JBI) Reviewer’s Manual for Scoping Review [20]. The review has been registered in the JBI Systematic Review Register with the title “A scoping review of factors influencing caring efficacy of direct care workers providing care to older people”. This proposed review will comply with the “Preferred Reporting Items for Systematic reviews and Meta-Analyses extension for Scoping Reviews (PRISMA-ScR)” checklist to facilitate complete and transparent reporting of results [21].

### Inclusion criteria

Inclusion criteria specify what is intended to be discussed in the review and the basis of inclusion of studies in the review [20, 22]. Clear congruency between the title, objective/s, review question/s, and inclusion criteria is required to perform a scoping review. The inclusion criteria for studies to be included in this review are classified by the type of participants, concept or the principal focus, context, and the type of studies as recommended by JBI reviewer’s manual for scoping review [20].

#### Type of participants

Studies that include direct care workers as study participants will be considered. Direct care workers in this study refer to all aged care staff providing direct care to older people living in aged care facilities. All paid/formal direct care workers will be considered in the review.

Studies will be excluded if their participants are as follows:

- Allied health workers (for example dietitian, exercise physiologist, occupational therapist, pharmacist, and social worker)
- Ancillary service providers in aged care (laundry and kitchen staff)
- Care workers in acute care or hospital settings
- Care workers providing home care services
- Informal caregivers such as family or relatives, unpaid caregivers, and volunteers, and
- Students

#### Concept

The key phenomenon of interest in this review is self-efficacy in caring. It refers to the belief or confidence of care workers in their ability to provide care to their clients. This review will exclude studies that discussed self-efficacy in providing only an individual component of care such as preventing falls, pain management, or managing agitated behaviors.

#### Context

The provision of aged care in residential aged care settings only will be included. Home care services will be excluded because studies have shown that the working environment and experiences of care workers in residential aged care settings were different from home care [23].

#### Types of sources

Any primary studies, including masters or doctoral dissertations, written in the English Language will be included. Studies will not be excluded based on the study design or date of publication.

This review will not include review articles, opinion pieces, book chapters or books, news articles, or studies conducted to develop measures of caring self-efficacy.

### Search strategy

The three-step search strategy will be followed based on the recommendation by JBI [20]. The first step involves a limited search of few databases to analyze text words in the title and abstract, as well as keywords and index terms that describe the article. Secondly, all databases considered for review will be searched to extract relevant studies using all terms identified in the first stage. Finally, the search for additional studies will also be carried out by scanning the reference list of relevant papers after full-text review.

Initially, CINAHL and MEDLINE have been selected in this review to identify index terms and keywords for searching for relevant studies. The search strategy has been developed in consultation with the senior library research advisor and has undergone peer review before finalization (see Additional file 2).

#### Information sources

An extensive search will be conducted using electronic databases—CINAHL, AgeLine, MEDLINE, PsycINFO, SCOPUS, and ProQuest Dissertations & Theses Global. The search strategy aims to locate both published and unpublished studies.

### Study selection

EndNote X9, a reference manager software program, will be used to remove duplicates from the list of studies identified by databases included in the review. The refined list of studies will be then imported into a Covidence to help in the management of the studies in the review [24]. Two reviewers will first screen five percent of the titles/abstracts independently based on the criteria for inclusion or exclusion of the studies for review and then discuss discrepancies to reach a shared understanding of the inclusion and exclusion criteria and refine the screening process before continuing for the final screening process of the title and abstract. Any disagreements in title/abstract screening will be resolved by the third reviewer. The full text of prospective articles will be then reviewed in detail by two reviewers separately. Any specific reasons for the exclusion of full-text studies will be recorded. The third reviewer will resolve the conflict but will also arrange a joint discussion with the review team in case of difficulty in deciding on the inclusion of the study in the review. The final decision will be made by consensus within the review team. The results of the search will be presented in the PRISMA-ScR flow diagram in the final report of the review [25].

### Data charting

Data charting will be carried out by two independent reviewers following the finalization of the studies to be included in this review. Data should be aligned with the objectives of the scoping review. The third reviewer will assess any discrepancies and discuss these with the review team to finalize data to be discussed in the result of the review.

Quantitative data will be extracted from quantitative studies and the quantitative component of mixed methods studies. Author(s), year of publication, year of study, source of data, research questions/objectives, study design and setting, study population and sample size, measures used, self-efficacy in caring, and potential factors influencing the self-efficacy such as sociodemographic factors, organizational factors, and other significant findings that relate to the review question and objectives will be extracted. Drafts of data charting forms for cross-sectional and intervention studies have been developed to record attributes and information of the studies relevant to the review objectives (see Additional file 3 and 4).

Qualitative data will be obtained from qualitative studies and the qualitative component of mixed methods studies. Characteristics of the included studies such as authors, aim of study, study population, gender, age range, methodological framework, and data collection and analysis method will be extracted. Similarly, participants’ quotes and researchers’ interpretation, statements, assumptions, and ideas related to review objectives will be extracted from the qualitative studies. A draft of data charting form has been developed for the review (see Additional file 5). Refinement of the data charting form is expected while carrying out the full review.

### Data analysis and presentation

The quantitative findings will be analyzed using descriptive statistics such as mean, frequencies, and percentages. The result will be presented in narrative form accompanied by the tabulated or charted results from selected studies. Descriptive qualitative content analysis will be carried out to analyze the qualitative findings. Qualitative data will be inductively categorized using computer software NVivo, version 12 [26]. Illustrative quotations will be presented to support the findings. The review team will discuss rigorously to analyze and present the results in a comprehensive manner.

## Discussion

A significant proportion of older people are receiving residential aged care in developed countries, and the number is increasing [9]. Quality of care to this population should be an important concern to their family members and aged care providers. Self-efficacy in caring has been identified as one of the factors that determine caring behaviors of care workers to their clients and eventually the quality of care [3, 6]. Identifying factors that influence the self-efficacy of aged care workers in caring could be a critical step in providing quality care to older people receiving aged care.

This scoping review aims to give an overview of the caring self-efficacy of aged care workers and identify factors that influence the caring efficacy of aged care workers. The inclusion of studies for this review will not be limited by the date of publication, source, or design of the study. Inclusion of grey literature as well as further search of the potential studies from the reference list of relevant studies will increase the comprehensiveness of this review. However, consideration of studies written in English only may limit the potential to understand self-efficacy of care workers from all parts of the world. Overall, the acquired result will provide documented evidence to policymakers and aged care providers to understand the self-efficacy of direct care workers in caring for older people and aid in identifying potential ways of enhancing their self-efficacy to improve their caring behaviors towards older residents living in residential aged care settings.

## Supplementary Information

**Additional file 1.** PRISMA-P.

**Additional file 2.** PsycINFO search strategy.

**Additional file 3.** Data extraction form for cross-sectional studies.

**Additional file 4.** Data extraction form for intervention studies.

**Additional file 5.** Data extraction form for qualitative data.

## Data Availability

Not applicable.

## Abbreviations

JBI: Joanna Briggs Institute
PRISMA-P: Preferred Reporting Items for Systematic reviews and Meta-Analyses for Protocols
PRISMA-ScR: Preferred Reporting Items for Systematic reviews and Meta-Analyses extension for Scoping Reviews
