# Supplementary material for: Self-efficacy of direct care workers providing care to older people in residential aged care settings: a scoping review protocol
Source: Syst Rev. 2021 Apr 10;10:105. doi: 10.1186/s13643-021-01655-z (PMC8035725; doi:10.1186/s13643-021-01655-z)
Supplement: Supplementary file 2 — Additional file 2. PsycINFO search strategy. [file 13643_2021_1655_MOESM2_ESM.doc]

**Search Strategy**

**Database:** APA PsycINFO <1806 to March Week 2 2020>

--------------------------------------------------------------------------------

1 exp Nursing Homes/ (8479)

2 exp Residential Care Institutions/ (42625)

3 exp Long Term Care/ (4999)

4 exp Assisted Living/ (706)

5 "residential facilit*".mp. [mp=title, abstract, heading word, table of contents, key concepts, original title, tests & measures, mesh] (2929)

6 ((extended or "long term") adj3 (care or facilit*)).mp. [mp=title, abstract, heading word, table of contents, key concepts, original title, tests & measures, mesh] (11557)

7 (("assisted care" or "assisted living" or "continuing care") adj3 (facilit* or institution*)).mp. [mp=title, abstract, heading word, table of contents, key concepts, original title, tests & measures, mesh] (666)

8 (residential adj3 care).mp. [mp=title, abstract, heading word, table of contents, key concepts, original title, tests & measures, mesh] (12644)

9 "aged care".mp. [mp=title, abstract, heading word, table of contents, key concepts, original title, tests & measures, mesh] (1230)

10 "institution* care".mp. [mp=title, abstract, heading word, table of contents, key concepts, original title, tests & measures, mesh] (1820)

11 exp Nurses/ (30832)

12 nurse*.mp. [mp=title, abstract, heading word, table of contents, key concepts, original title, tests & measures, mesh] (71926)

13 exp Nursing/ (21786)

14 "nursing staff".mp. [mp=title, abstract, heading word, table of contents, key concepts, original title, tests & measures, mesh] (5143)

15 (assistant* adj2 nursing).mp. [mp=title, abstract, heading word, table of contents, key concepts, original title, tests & measures, mesh] (708)

16 (nursing adj3 personnel).mp. [mp=title, abstract, heading word, table of contents, key concepts, original title, tests & measures, mesh] (575)

17 "care aide".mp. [mp=title, abstract, heading word, table of contents, key concepts, original title, tests & measures, mesh] (22)

18 "care attendant*".mp. [mp=title, abstract, heading word, table of contents, key concepts, original title, tests & measures, mesh] (36)

19 "paid carer*".mp. [mp=title, abstract, heading word, table of contents, key concepts, original title, tests & measures, mesh] (97)

20 "nursing home aide*".mp. [mp=title, abstract, heading word, table of contents, key concepts, original title, tests & measures, mesh] (17)

21 "nurse aide*".mp. [mp=title, abstract, heading word, table of contents, key concepts, original title, tests & measures, mesh] (144)

22 "care assistant*".mp. [mp=title, abstract, heading word, table of contents, key concepts, original title, tests & measures, mesh] (218)

23 "support worker*".mp. [mp=title, abstract, heading word, table of contents, key concepts, original title, tests & measures, mesh] (739)

24 "care staff*".mp. [mp=title, abstract, heading word, table of contents, key concepts, original title, tests & measures, mesh] (2654)

25 "formal carer*".mp. [mp=title, abstract, heading word, table of contents, key concepts, original title, tests & measures, mesh] (37)

26 "care worker*".mp. [mp=title, abstract, heading word, table of contents, key concepts, original title, tests & measures, mesh] (5069)

27 exp self-efficacy/ (22150)

28 exp Competence/ (25172)

29 exp Professional Competence/ (10421)

30 exp self-confidence/ (3532)

31 efficacy.mp. [mp=title, abstract, heading word, table of contents, key concepts, original title, tests & measures, mesh] (146111)

32 competen*.mp. [mp=title, abstract, heading word, table of contents, key concepts, original title, tests & measures, mesh] (100813)

33 confiden*.mp. [mp=title, abstract, heading word, table of contents, key concepts, original title, tests & measures, mesh] (93653)

34 "nurs* skill*".mp. [mp=title, abstract, heading word, table of contents, key concepts, original title, tests & measures, mesh] (358)

35 1 or 2 or 3 or 4 or 5 or 6 or 7 or 8 or 9 or 10 (56843)

36 11 or 12 or 13 or 14 or 15 or 16 or 17 or 18 or 19 or 20 or 21 or 22 or 23 or 24 or 25 or 26 (87427)

37 27 or 28 or 29 or 30 or 31 or 32 or 33 or 34 (327548)

38 35 and 36 and 37 (1089)

39 limit 38 to English (1051)

***************************
